# Alkali–Silica Reactions: Literature Review on the Influence of Moisture and Temperature and the Knowledge Gap

**DOI:** 10.3390/ma17010010

**Published:** 2023-12-19

**Authors:** Olusola D. Olajide, Michelle R. Nokken, Leandro F. M. Sanchez

**Affiliations:** 1 Department of Building, Civil & Environmental Engineering, Concordia University, Montreal, QC H3G 1M8, Canada; olusola.olajide@concordia.ca (O.D.O.); m.nokken@concordia.ca (M.R.N.); 2 Department of Civil Engineering, University of Ottawa, Ottawa, ON K1N 6N5, Canada

**Keywords:** alkali–silica reaction, moisture, relative humidity, temperature, damage rating index, relative humidity threshold

## Abstract

The alkali–silica reaction is a universally known destructive mechanism in concrete that can lead to the premature loss of serviceability in affected structures. Quite an enormous number of research studies have been carried out focusing on the mechanisms involved as well as the mitigation and prevention of the reaction. A few in-depth discussions on the role of moisture and temperature exist in the literature. Nevertheless, moisture and temperature have been confirmed to play a vital role in the reaction. However, critical assessments of their influence on ASR-induced damage are limited. The available moisture in concrete needed to initiate and sustain the reaction has been predominantly quantified with the relative humidity as a result of difficulties in the use of other media, like the degree of capillary saturation, which is more scientific. This paper discussed the current state of understanding of moisture measurement in concrete, the role of moisture and temperature in the kinetics of the reaction, as well as the moisture threshold needed for the reaction. Furthermore, the influence of these exposure conditions on the internal damage caused by ASR-induced deterioration was discussed.

## 1. Introduction

### 1.1. Background

The alkali–silica reaction (ASR) has been a menace to the durability of concrete infrastructure since its discovery several decades ago [1]. The ASR is a chemical reaction that results from the interaction between reactive silica and alkalis in a humid environment; this reaction generates a gel that swells upon moisture uptake, leading to induced expansion and cracking in the affected concrete. The occurrence of ASR depends on many factors and although the failure of ASR-affected structures is not common, it can induce several durability and serviceability issues [2]. Extensive research has been conducted on the mechanism of the ASR and the development of maintenance strategies [1,2,3]. However, in many cases, locally available aggregates exhibit some level of reactivity, necessitating the need to prevent the reaction. Unfortunately, there is currently no known technique to completely prevent the reaction once all the required conditions are met. Nevertheless, supplementary cementitious materials (SCMs) [4,5,6,7] and lithium compounds [8,9,10,11] have often been incorporated into new concrete structures to slow down the reaction. However, due to the reported shortages in the supply of SCMs like fly ash [12] and the cost of using available admixtures [9], there is an urgent need to explore additional SCMs or admixtures that could be used [13,14]. In existing structures already affected by ASR, there is no known method to halt the reaction. Common mitigation measures include controlling moisture availability through the application of coatings/sealers [15,16], the impregnation of lithium, the release of stresses through slot cutting, and external restraint by post-tensioning [17]. However, new cracks have been reported to appear years after the application of such techniques [18,19]. Given the challenges posed by the unavailability of non-reactive aggregates, especially in areas prone to ASR; diminishing SCMs; the availability of high-alkali cement; and limitations to existing coating systems; exploring the influence of moisture and environmental conditions like temperature remains crucial for preventing the ASR and selecting effective maintenance strategies.

Moisture is an important factor for the ASR and its availability in concrete has been often appraised with the aid of relative humidity (RH), which is not the true indicator of available water in concrete. Other methods like the degree of capillary saturation, chemical reactions, electrical resistance, etc., have also been used. Nonetheless, various researchers have reported numerous RH thresholds for the onset of the reaction. Some conclusions were based on the external RH, while others were based on the internal RH, i.e., relative humidity outside or inside the concrete, respectively [3,20,21]. Furthermore, some authors have claimed the threshold is dependent on the temperature [22], while others have suggested that the reactivity level of the aggregates is paramount [3,23]. Some of these conclusions were inferred from tests using reactive fine aggregates [24] and others with reactive coarse aggregates [25]. Other factors, such as size, drying and wetting cycles, etc., can also affect the availability of moisture in concrete and, subsequently, the kinetics of the reaction.

Temperature plays a pivotal role in the ASR as with other durability problems in concrete. Concrete exposed to high temperatures is more likely to experience the ASR as a result of the increase in the rate of expansion aided by the rapid dissolution of silica and the diffusion of ions. However, temperature could also play a counter role by improving drying and thereby reducing the absorption of water by ASR gel [26]. Furthermore, temperature can significantly affect the relative humidity of the air that concrete is exposed to; the higher the temperature, the higher the capacity of the air to take in more moisture, thereby reducing the relative humidity. The impact of this relationship and its influence on ASR-induced expansion are yet to be well studied.

**Figure 1:**
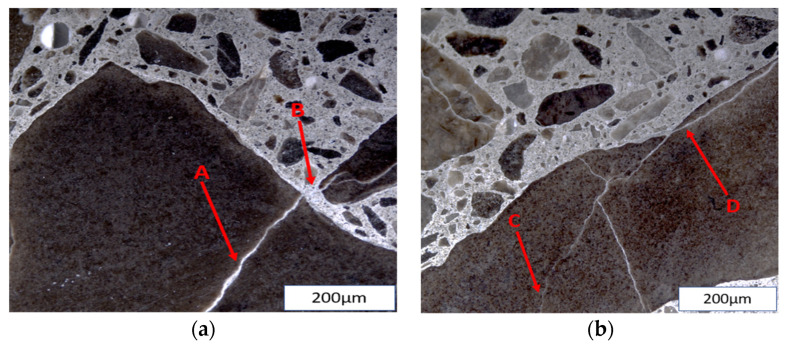
(**a**) A (Open crack in aggregate with reaction product; OCAG), B (Crack in cement paste with reaction product; CCPG); (**b**) C (Closed crack in aggregate; CCA), D (Open crack in aggregate; OCA).

The ASR is initiated in the concrete microstructure and produces internal cracks long before the manifestation of surface cracks or other symptoms. The internal cracks in ASR-affected concrete can be correlated to its induced expansion [27], thus emphasizing the need to assess the influence of various exposure conditions on the microstructural behaviour of the reaction. Cracks due to ASR, as shown in Figure 1, were observed as being initiated within the aggregate particles (i.e., fine or coarse) and propagating to the cement paste. Coarse aggregates have been known to have a different effect on induced expansion when compared to fine aggregates [28]; cracks generated by the latter propagate faster into the cement paste due to their small size [29]. Thus, the physical integrity determined by the inner damage (pattern and extent of cracks) of affected concrete at different moisture and temperature conditions, bearing distinct aggregates with different reactivity levels, can vary.

This paper aims to review the influence of moisture and temperature on the ASR. To provide a comprehensive overview, the available methods for measuring moisture in concrete, the moisture threshold needed for the reaction, the influence of cyclic conditions, and the multifaceted role of temperature in the ASR will be discussed. While the influence of these conditions in the kinetics of ASR is well known, existing studies have primarily focused on expansions recorded over time. This study seeks to broaden the discussion by exploring the evaluation of damage due to the ASR under various moisture and temperature conditions using microscopic assessment.

### 1.2. The Mechanism of the ASR

**Figure 2:**
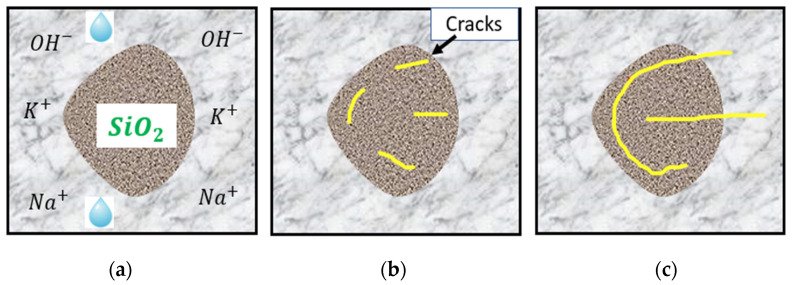
Sequence of ASR: (**a**) factors needed for ASR; (**b**) initiation of cracks; (**c**) propagation of cracks. Modified from [31,32].

In the presence of adequate moisture, the alkalis present in cement—mainly sodium and potassium oxides—react with poorly crystallized siliceous phases in aggregates. According to Juliana et al. [30], the high alkalinity of concrete pore solutions promotes the exchange of hydroxyl ions ($OH^{-}$) with either sodium or potassium through an ion exchange process, and calcium-rich ASR gel is formed when amorphous or poorly crystallized silica is attacked. The ASR gel formed has a great affinity to water and swelling and exerts pressure in all directions. This mechanism leads to stresses which, in turn, generate cracks that initiate from the aggregates and propagate into the cement paste, as schematically shown in Figure 2.

The hydroxyl ions ($OH^{-}$) present in the pore solution from the hydration of cement attack the silica in aggregates, leading to the decomposition of the silica structure. The $OH^{-}$ disrupt the siloxane bonds (≡Si–O–Si ≡) and break the network to form a silanol group (≡Si–OH). The progressive attack of the hydroxyl ions on the silanol group (≡Si–OH) results in the network dissolution of silica. According to Rajabipour et al. [1], this dissolution can be hastened with an increase in temperature and/or alkalinity. The high alkalinity of the pore solution promotes the exchange of hydroxyl ions (OH^−^) from the silica surface with alkali ions, such as sodium (Na^+^) or potassium (K^+^), in the pore solution [33]. The presence of alkali ions triggers the formation of an ASR gel composed of alkali silicates. The reaction mechanism is time-dependent [34]; at the inception of the reaction, the ASR gel is deficient in calcium. However, as the reaction progresses, the dissolution of solid portlandite (Ca${\left(OH\right)}_{2}$) in the cement occurs. Although the role of calcium ions remains controversial [35], popular claims remain stating that calcium hydroxide (Ca${\left(OH\right)}_{2}$) serves two primary roles: it functions as a “buffer” by helping to sustain a high pH level, which corresponds to a significant concentration of hydroxyl ions within pore solutions [36], and allows for the potential exchange of Ca++ ions with alkali ions in the gel and forcing the return of the alkali ions into the pore solution, a phenomenon referred to as alkali recycling. Despite the controversial state of the role of calcium ions in the reaction, their availability remains vital for the expansive behaviour of the gel. Irrespective of this, ASR gels have been known to possess a low Ca/Si- ratio compared to C–S–H gel. The presence of Ca in the gel ensures its high viscosity and swelling upon the imbibition of water. Otherwise, the gel dissolves and remains in the solution [37].

The deficiency of calcium in the concrete pore solution will further lead to the disintegration of more portlandite from the cement. This, combined with the presence of alkali ions in the pore solution, initiates a repetitive sequence, ultimately leading to the formation of additional alkali–silica gels. Buttressing the time dependency of the reaction, the reaction can be distinguished into two stages [38]. The first stage is composed of amorphous ASR products that are generated in the aggregates in proximity to the cement paste. The products move into the interior of the aggregates, generating pressure that results in cracks. Since the reaction is a continuous one, new ASR products formed in the second stage are crystalline in structure, filling the existing cracks and exerting swelling pressure that propagates the cracks into the cement paste. There exist several other analogies in the literature about the ASR gel swelling mechanism [39,40,41]. Nonetheless, the reactivity is endless and will continue until sufficient alkalis, calcium ions, siliceous phases, and moisture are no longer available [3].

## 2. Review Objectives

The objective of this research was to conduct a thorough examination of the impact of moisture and temperature on the alkali–silica reaction (ASR). This review highlights the variations in the existing literature concerning the role of moisture in the reaction, as well as the limited availability of articles specifically addressing the influence of moisture and temperature on the microstructural properties of ASR-affected concrete.

## 3. Moisture in Concrete

Moisture plays a vital role in the durability of concrete [42]. Its availability beyond a specific limit could be favourable for the alkali–silica reaction, freezing and thawing [21], carbonation [43], etc. Available water in concrete can be categorized into chemically bound water, physically bound water, and capillary (free) water [44]. Physically bound and free water are responsible for the transportation mechanism in concrete pore structures, and their distribution in this space is influenced by the moisture content, while chemically bound water is non-evaporable and fixed in the hydrate phases [45]. The moisture content available in concrete can therefore be interpreted as the volumetric ratio of the sum of physically bound and free water to the pore space [44]. This moisture content has been measured using several methods.

The gravimetric method is the most dependable and accurate technique for measuring moisture in concrete, especially when representative samples can be collected from the body under investigation. However, the destructive nature of the test renders it unsuitable in some circumstances [46]. Some other relevant methods are itemized in Table 1, but most of these methods do not measure moisture in concrete directly, thus limiting their use. Other challenges include cost, calibration challenges, and ease of usage. Nevertheless, the relative humidity and degree of capillary saturation have been popularly used for such measurements.

The choice of relative humidity or degree of capillary saturation measurement is dependent on the type of moisture (water vapour or liquid water) under consideration. The assessment of frost damage in concrete can be further emphasized with the use of the degree of capillary saturation, which illustrates the number of filled pores. Whereas, in describing concrete deterioration involving chemical reactions influenced by the activity of moisture in the pores, the relative humidity measurement can be more efficient [55].

The RH considers the amount of water vapour in the air. The internal RH of early-age concrete is hugely dependent on the water-to-cement ratio (w/c), and the readings can be obtained with hygrometers [56]. The RH is assumed to be 100% at complete saturation after casting (liquid-like condition). According to Zhang et al. [57], this condition exhibits a continuous liquid network, and the RH of the air is close to 100% [58]. After some time, the RH starts dropping due to hydration and/or evaporation. As hydration continues, the formerly continuous liquid network gets breached, secluding the pore water and leading to the decrement in the internal RH.

The degree of capillary saturation (DCS) of concrete, on the other hand, can be obtained through the ratio of the volume of water available in the concrete to the volume taken up when the concrete is subjected to capillary suction until equilibrium is reached [55]. A handful of strategies have been adopted to measure the DCS in the literature, most of which are focused on obtaining the weights of the sample before and after drying as well as the weight after capillary water absorption, which can be achieved by submerging the sample or with one surface of the sample in contact with water [59,60]. The procedure (weighing and drying) for DCS can limit its use for in situ measurements, unlike that for RH [61,62].

The DCS and RH focus on different properties of moisture in concrete and are affected by distinct factors. However, there exists a seemingly linear relationship between them. According to a study by Weiss [63] on concrete specimens with a w/c of 0.42, the degree of saturation increases as the RH increases. Despite the challenges involved in performing the DCS, this test seems to be more scientific; the actual amount of water in concrete can be determined. However, the RH is more suitable for use in ASR-affected concrete due to the ease of measurement. This can be performed easily with the aid of sensors. Furthermore, the ability to carry out RH measurements in situ, especially in the laboratory, without interfering with the ASR-accelerated performance tests through the drying of samples and the capillary water absorption procedures required for DCS makes the RH more suitable.

## 4. The Role of Moisture in the Alkali–Silica Reaction

Moisture is a critical factor for the initiation and sustenance of the ASR [64]. The moisture present in ASR-affected concrete is largely due to the mixing water (w/c) and ingress of water from external sources. Such ingress could be from rain, ambient environmental conditions, drainage, etc., or a combination of these.

The role of moisture in the reaction has been well documented [65]. Its availability serves as a transportation medium for the ions involved in the reaction and for the formation/swelling of the resulting ASR gel. The transportation of ions can be further enhanced in a highly porous and permeable medium obtainable in concrete with high w/cm [66]. As a result, the reduction in the available moisture has been used to mitigate the reaction. On the other hand, a reduction in the w/cm could potentially aid the reaction. During hydration at low w/cm, the products are less heterogeneous and less portlandite is produced. Hydroxyl ions are dissolved from the Ca(OH)_2_ in order to maintain equilibrium with the high alkali concentration in the pore solution as a result of the release of Na^+^ and K^+^ from the cement, leading to an abundance of ions in the pore solution [60]. According to Nilsson and Peterson [67], the reaction can be initiated even at a low RH of 80%, but progresses slowly due to the low rate of diffusion. The ASR gel swelling that can arise in this condition may not be sufficient to exert significant pressure on the surrounding paste. If the concrete is re-exposed to water or moved to a more saturated condition, the rate of the reaction is improved, and expansion is significantly improved [68,69].

The moisture needed for the ASR has been prominently measured using the RH [3,22,70]. Poyet et al. [22] examined the kinetics of the ASR and resulting expansion in cylindrical mortar samples stored in different external relative humidity conditions and, based on their studies, ASR expansion increases as RH increases. Few researchers have argued that the degree of DCS is a better means of moisture measurement for the reaction [55,71]. Nonetheless, damage due to the ASR intensifies as the DCS increases [55], and a DCS threshold of 90% has been reported to be needed [71].

### 4.1. Relative Humidity Threshold

The availability of a sufficient amount of water has been confirmed as a crux for the reaction [26]. Based on the discussion in the previous sections, no significant distress can be recorded at low moisture conditions. As a result, the concept of the RH threshold has been introduced for the prevention and maintenance of ASR-affected structures.

The absorption of water and expansion of the ASR gel are dependent on the RH. According to a work carried out by Bažant and Steffens [72], the absorption capacity of the gel greatly diminishes when the RH of the air in the pores falls below 85%. Furthermore, expansion is impeded at low RH levels. According to the studies in [73], no expansion was recorded when mortar samples were stored at 65% RH and 38 °C, while considerable expansion was reported in those stored at similar temperatures but at 85% RH, buttressing the claims that a minimum RH of 80–85% is needed for the reaction [74]. Some other authors have posited a threshold of 80% [21,67]. Yet, numerous RH thresholds exist in the literature. Olafsson [70] reported 80% at 23 °C, which falls to 75% at 38 °C, while Ludwig [20] reported it to be between 80–85% at a lower temperature of 20 °C.

**Figure 3:**
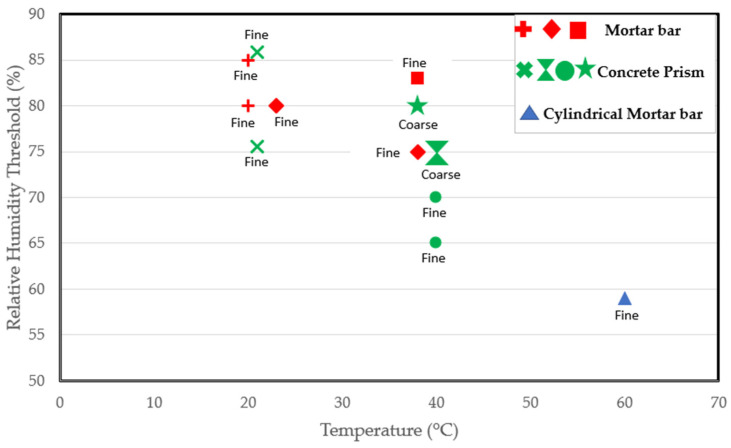
Relative humidity threshold as a function of temperature, specimen type, and reactive content from several studies [3,20,22,23,25,70,75,76].

Some researchers have worked at higher temperatures. Tomosawa et al. [25] and Kurihara and Katawaki [24] investigated the reaction at 40 °C and reported a threshold of 75%. Poyet et al. [22] reported a threshold of less than 59% at 60 °C as an expansion of 0.06% was recorded in cylindrical concrete specimens exposed to 60 °C after just 100 days. Thus, the RH threshold could depend on the temperature [22]. As indicated by Figure 3, which includes various studies on RH thresholds at different temperatures, it is evident that the moisture needed for the reaction at room temperature surpasses that required at the standard conditioning temperature of 38 °C. Moreover, the moisture requirement is even lower at a higher temperature of 60 °C. Consequently, the critical RH for ASR-induced expansion decreases with increasing temperature, confirming the claims in [22]. However, in a report by Pedneault [23], the author stated that the critical RH depends on the form of siliceous minerals of the aggregate. Given the different reactivity levels of aggregates, some aggregates are expected to have a faster rate of expansion. As a result, very highly reactive aggregates could expand at a moisture level where moderately reactive aggregates would not. Furthermore, the reactive content (fine versus coarse) of the mix could influence the kinetics of the reaction, as clearly shown in Figure 3.

According to the studies [25,75] carried out on concrete prisms stored at 40 °C, as shown in Figure 3, specimens containing reactive coarse content have an RH threshold of 75% compared to the 65–70% RH needed for the mix prepared using a reactive fine aggregate. Hence, a lower moisture level could be sufficient to initiate the reaction in specimens involving reactive fine aggregate. After all, ASR-induced cracks propagate faster in reactive fine aggregates due to their small size. Lastly, the RH threshold required for the reaction could vary depending on the sample size and shape. After all, the rate of the reaction and ultimate ASR-induced expansion differ in concrete prisms, cubes, and cylinders incorporating reactive aggregates [77]. Thus, the varying sample types used in the available studies may play a role in the varying thresholds reported in the literature.

### 4.2. The Role of Alternate Drying and Wetting on the Availability of Moisture for the Alkali–Silica Reaction

Concrete exposed to natural environmental conditions is subjected to several drying and wetting cycles during its service life; these conditions have an influence on the available moisture in the concrete and the development of the ASR. According to Zhang et al. [58], when concrete is exposed to moisture (wetting), the interior RH increases rapidly within a short period of time and then reaches a stable level; this, however, is dependent on the concrete grade, permeability, size, and other properties of hardened concrete; duration of the wetting cycle; etc. Conversely, during drying, the interior RH reduces gradually [58]. According to the study by Zhang et al. [58], concrete cubes with 28 days of compressive strength of 30 MPa were subjected to a dry and wet cycle (14 and 7 days, respectively) and the internal RH at the centre of the 60 × 100 × 350 mm cubes of the samples was measured. As expected, the RH reduced during the drying cycle but failed to reach the initial moisture level when subjected to wetting. This could have been due to the duration of the wetting cycle. As a result, such a phenomenon could influence the available moisture in concrete for the ASR [15,78].

To discuss the influence of the cyclic condition on ASR, Stark et al. [26] confirmed that cyclic wetting and drying conditions could stimulate the initiation and sustenance of the reaction. Of course, there was less moisture to be absorbed by ASR gel during drying; nevertheless, the increase in temperature improved the concentration of hydroxyl ions in the pore solution, which exacerbated the attack on the poorly crystallized aggregates. As a result, expansion progressed exponentially in the following wetting cycle. Furthermore, in the next dry period, more non-expansive reaction products were formed and drying increased the extent of the cracks formed in the prior wetting cycle. Thus, minimizing wetting and drying cycles is vital in controlling the reaction [79].

Evidently, expansion due to the ASR is significantly retarded in the drying cycle, especially towards the surface of the concrete. The reaction can, however, progress actively in the inner depth but not close to the outer surface due to the moisture gradient. According to Kagimoto and Kawamura [80] in their study on lab-conditioned concrete specimens, a relative humidity of up to 80–90% (a moisture level sufficient to initiate the ASR) was measured in the middle of the concrete cylinders during drying; such variations in expansion across the depth of affected concrete were responsible for surface cracks due to the ASR [81]. A similar observation was recorded in field samples by Stark et al. [26]. It is, however, worth noting that this phenomenon could be dependent on the size of the concrete member. Slender members would experience a rather quicker drop in RH across their depth.

## 5. The Role of Temperature in the Kinetics of the ASR

Increases in moisture, temperature, alkalinity, or a combination of any of these factors have been adopted in the laboratory to accelerate the rate of ASR-induced expansion, among which, increases in temperature have been reported to be the most efficient [82]. Although temperature has not been noted as one of the primary factors (alkalis, silica, water) needed for the ASR, its influence can affect the availability of these factors. Its influence extends even further into the structure of the gel produced upon the initiation of the reaction. An increase in temperature has proven to be effective in enhancing the dissolution of silica, thereby accelerating the formation of ASR gel [83,84]. The influence of temperature on the accelerated dissolution of silica was reported in [85]; the authors recorded an increase in the rate of dissolution of siliceous particles from 71% to 100% when their storage temperature was increased from 25 °C to 80 °C. The role of temperature on the availability of alkalis in concrete pore solutions can be categorized into two groups. An increase in temperature enhances the mobility of the alkali ions (K and Na); aside from that, the concentration of ions in concrete pore solutions increases at elevated temperatures. On the other hand, some reactive aggregates have been found to contribute alkalis to concrete pore solutions, and temperature has been reported to affect the release of such additional alkalis. In [77], elevated temperatures resulted in a greater total alkali content released from the three tested aggregates (Sudbury, Spratt, and Springhill) into the alkaline solutions at three different temperatures (21 °C, 38 °C, and 60 °C), with the highest release occurring at 60 °C. An exponential increase in alkali release was reported when the temperature was increased from 20 °C to 80 °C [86]. It is, however, worth noting that after considering other studies in the literature [87] and the comprehensive table of available works in the literature on aggregates and alkali release put together in [88], the amount of alkali release can also be influenced by the mineralogy of the aggregates and the type of aggregate (coarse versus fine).

The improved properties due to elevated temperature can influence the properties of the resulting ASR gels. As noted in the study on synthesized gel in [89], gels at elevated temperatures and higher molarities exhibit notable differences in both their microstructure and macrostructure when compared to those generated under milder conditions. For instance, the structure of the crystalline product formed at 38 °C/40 °C was different from that formed at 60 °C using the Raman spectra [38]. The differences in the mobility/dissolution parameters and structure of the gels can influence the kinetics of the reaction, leading to a possible rise in the expansion rate by 1.7 times when the temperature is increased from 38 °C to 50 °C [82]. As a result, several laboratory performance tests were conducted at high temperatures to increase the rate of expansion and reduce their duration [90,91,92]. Table 2 comprises a database of several ASR tests that have been conducted at different temperatures, highlighting the uniqueness of each test. The table includes the test conditions, durations, and ultimate expansion results. Furthermore, the 28-day expansion results are highlighted to show the influence of temperature on the evolution of the reaction over time.

**Figure 4:**
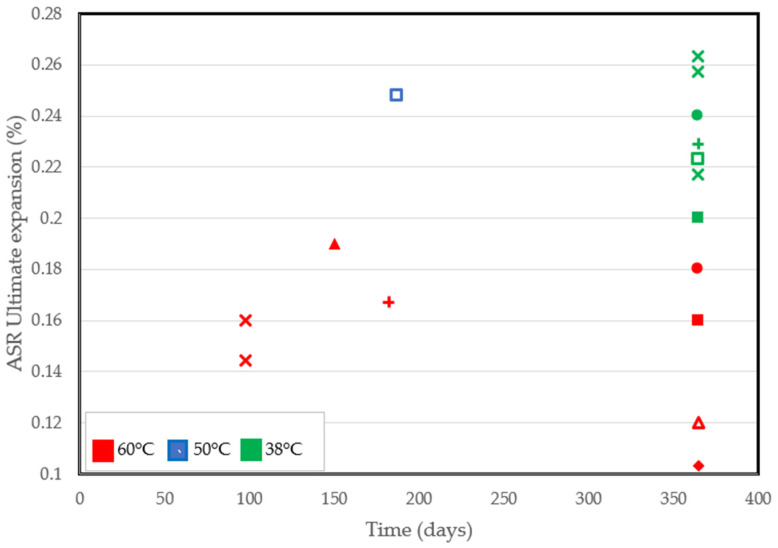
ASR-induced ultimate expansion at different temperatures reported by several authors [71,77,82,88,90,91,92,93].

Despite the pros of the accelerated performance tests conducted at high temperatures, some shortcomings have been identified. As a matter of fact, the ultimate expansion can be lower at high temperatures [82,90,93]. Significant reductions in the ultimate expansion recorded from several accelerated tests are in Figure 4. At similar ages of exposure, specimens conditioned at 38 °C all had a higher final expansion than at 60 °C, even in tests involving the use of similar aggregates. Based on findings from the literature, increased alkali leaching, non-reactive sand, improved porosity, and reduced calcium content [90,96] are factors that could be deemed responsible for a lower final expansion.

The curing temperature to which concrete specimens are exposed before being conditioned for the ASR can affect their expansion. High curing temperatures tend to result in a high degree of hydration, consequently reducing the porosity of concrete and thereby reducing water uptake properties and the mobility of ions [60]. Although this does not stop expansion, the final expansion can be much lower [71]. Considering this, some researchers [97] developed a micro-mechanical expansion model that assessed the effect of ASR gel in cement paste in terms of hydrostatic pressure in the microstructure of cement at different temperatures. Porosity is considered an important characteristic of concrete microstructure, and experimental results prove its reduction at high temperatures because of hydration. According to the authors, the hydrostatic pressure decreases with increases in temperature due to improved porosity, leading to a reduced diffusion of alkali and ASR expansion. However, since hydration is a continuous process, the rate of expansion could be higher at early ages and dwindle at later ages.

An increase in temperature can indirectly lead to a reduction in calcium concentration and accelerate various chemical reactions, including the dissolution of alkali compounds and their mobility, thus enhancing the concentration of the alkalis in the pore solution [38] and the ASR reaction. The absorption of Ca by existing gel reduces the concentration of Ca ions in the pore solution. Hence, new ASR gels generated may lack the required expansive properties that can be acquired by reactions with Ca ions. In fact, a less viscous gel has been reported to be produced in such conditions that flows into the concrete matrix without an accompanied swelling pressure [60,94]. In the same context, several authors have reported the instability of ettringite at high temperatures greater than 60 °C leading to the presence of sulfate ions and aluminium in the pore solution [98]. The presence of these ions lowers the concentration of Ca and, consequently, the ASR expansion.

Other factors like the drying of the ASR gel leading to the reduced absorption of water by the gel [26] and alkali leaching have been studied [93,98,99,100,101]. The role of alkali leaching has been the most studied from the above factors. The existing literature on notable possible solutions includes the adoption of a maximum testing temperature of 50 °C/60 °C [82], wrapping of test samples [60], and reduction of the testing duration [87]. Nevertheless, most of these solutions have proven to be abortive. Of notable importance is the wrapping of concrete specimens during testing. For instance, in the study carried out by Kawabata et al. [93], concrete prisms containing highly reactive aggregates wrapped with alkaline cloth to reduce alkali leaching were conditioned to 20 °C, 40 °C, and 60 °C in accordance with [99]. The correlation of the results was confirmed by calculating the total alkali content in the wrapping cloth and assessing the water at the bottom of containers. The kinetics of the reaction differed at different temperature levels and no linear relationship existed between the temperature and ultimate expansion; specimens at 60 °C yielded the highest early expansion, but the highest final expansion was achieved at 40 °C, which was only slightly higher than the ultimate expansion at 20 °C. Since this method [99] made provisions for the recycling of alkalis, other factors that have been previously discussed or combinations of these factors might be hypothesized to be responsible for the low ultimate expansion at 60 °C.

## 6. The Influence of Moisture and Temperature on the Microstructure Properties of ASR-Affected Concrete

The ASR is known to induce the degradation of the microstructure of concrete before its appearance on the macroscale. This facilitates the need to understand the behaviour of the mechanism from a microstructural point of view. Several microscopic methods have been established for such assessment, some of which are highlighted in Table 3. The scanning electron microscope has been the most used tool; this has often been coupled with other tools like the EDS and XRF among others to assess the properties of ASR gel and induced cracks. Other tools such as the TEM and CT scan are also becoming popular for the diagnosis of the ASR. It should be noted that while some of the assessments from these tests are quantitative, they are somewhat relative [93]. Furthermore, these tools have been proven reliable in determining the cause of deterioration (the ASR, in this case), and visual descriptions of the extent of damage can be made. However, a quantitative output for assessing the extent of damage is lacking. The damage rating index (DRI) has so far proven reliable in determining the cause (using the location, features, and propagation of cracks) and extent of damage in concrete (using the DRI number) [102,103,104].

The DRI is a petrographic-based semi-quantitative analysis performed on a polished surface using a stereomicroscope with a magnification of 15–16x, and damage features—as highlighted in Table 4—are counted in a 1 cm^2^ square gird. The count of each feature is multiplied by the corresponding weighting factor. The DRI number is obtained by taking the sum of the weighted features and normalizing it to 100 cm^2^ [112].

**Figure 5:**
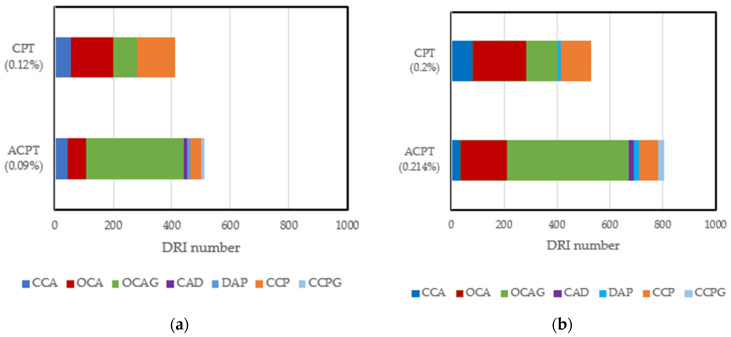
Damage features counted in CPT (38 °C, >95% RH) and ACPT (50 °C, >95% RH) at 0.1% and 0.20% expansion levels: (**a**) comparison of DRI results at 0.1% expansion; (**b**) comparison of DRI results at 0.2% expansion. Adapted from [16,66].

Quite a huge amount of work has been conducted to evaluate the reliability of the DRI in assessing damage due to ASR and other internal swelling reactions. In summary, the DRI number has been reported to increase as ASR-induced deterioration increases [27]. Cracks in aggregates have been categorized into two types, namely, sharp cracks and onion skin cracks [113]. Furthermore, a qualitative model for crack propagation in ASR-affected concrete has been developed: it highlights the development of several single sharp and onion cracks in aggregates at low expansion levels. These cracks increase in length, connect, and propagate into the cement paste at moderate to high expansion levels [112]. Despite the enormous work that has been conducted with this tool, most studies have been limited to concrete/mortar samples conditioned at high and constant moisture levels. No study has been carried out to directly evaluate the influence of moisture on ASR-induced damage features. An assessment of the few microstructural studies [32,82] that have been conducted at different temperatures shows that an increase in temperature can lead to changes in the pattern, location, and number of cracks, even at similar expansion levels. Comparing the DRI results of samples affected by the ASR through the accelerated concrete prism test (ACPT) carried out [82] at 50 °C and the concrete prism test (CPT) carried out [32] at 38 °C, at ~0.1% and 0.2% expansion levels, a higher DRI number was recorded in the ACPT than in their counterparts in the CPT. Figure 5 combines the data from published sources and shows that the damage features are different; more open cracks in aggregates with reaction products (OCAG) were counted in ACPT than in CPT at similar expansion levels. This is because of the fast rate of expansion leading to more induced cracks. Another interesting point is the influence of temperature on the number of cracks in the cement paste at similar expansion levels. Fewer CCPs were recorded at 60 °C compared to 38 °C. This interesting result validates the hypothesis that exposure conditions can influence the microstructural properties of ASR-affected samples, even at similar expansion levels. Since this comparison is based on ASR-induced expansion and DRI results from two different authors, research is needed to improve our understanding of the influence of these exposure conditions on internal damage caused by ASR-induced expansion.

## 7. Conclusions

- Moisture is an important factor for the reaction and an ample amount is needed. The utilization of relative humidity, an indirect technique measuring the quantity of water vapour in the air, has been both commonly employed and scrutinized. However, it has been preferred as a means of moisture measurement in ASR-affected concrete due to the ease of measurement compared to other methods like the degree of capillary saturation.
- A moisture threshold of 80% RH has been generally agreed to be required to initiate and sustain the reaction. However, based on reviewed works in the literature, there exist studies that report thresholds lower and higher than this value. The threshold could be dependent on the reactivity of the aggregate, temperature, or other factors that differed among the existing studies.
- Concrete subjected to wet and dry cycles are prone to exhibiting moisture gradients, and their impact on ASR expansion may vary depending on the size of the concrete member, with slender members experiencing a quicker drop in RH across their depth. In managing the reaction, minimizing the number of wetting and drying cycles is critical. Furthermore, understanding the combined influence of moisture availability, cyclic conditions, and concrete properties is essential for developing effective strategies to mitigate the deleterious effects of the ASR.
- The influence of temperature on the ASR is multifaceted and extends beyond its direct inclusion among the primary factors (alkalis, silica, moisture) necessary for the initiation and continuation of the reaction. An increase in temperature has been identified as the most efficient factor for accelerating ASR-induced expansion in laboratory conditions. However, the ultimate expansion can be lower at higher temperatures. Efforts to address these complexities have not consistently proven to be effective. Hence, the relationship between temperature and ASR-induced expansion is complex and non-linear.
- The assessment of the ASR in concrete has evolved to include a detailed assessment of the influence of induced expansion on microstructural properties, recognizing that ASR induces microstructural degradation before visible signs appear at the macroscale. This shift in focus emphasizes the need to comprehend the influence of moisture and temperature in ASR-affected concrete from a microstructural standpoint rather than just the induced expansion. The availability of moisture and temperature has been reviewed to have a possible influence on the properties of cracks induced by the ASR.

## 8. Recommendations

In order to advance our knowledge of the influence of moisture and temperature on the ASR, the recommendations highlighted hereafter are to be considered:

- The ambiguity surrounding the behaviour of the ASR due to variations in constituents and exposure conditions results in uncertainties when comparing studies. These variations hinder the establishment of a universal RH threshold. Different thresholds have been reported by authors and it is likely that the threshold is influenced by factors such as temperature, aggregate reactivity, and the sample sizes used to determine reported thresholds. Further research is required to determine the most important influencing factor.
- Existing studies failed to consider the effect of initial moisture content/internal RH in determining the critical RH for the ASR. Considering this effect on the evolution of RH in affected concrete over time would further our understanding of the reaction.
- Temperature has been proven to enhance the rate of ASR-induced expansion; however, there could be a reduction in ultimate expansion due to factors like alkali leaching, the drying of gel, and improved pores. This can influence the assessment of the full reactivity potential of aggregates being assessed. Thus, more research is needed to develop fast and reliable tests that can withstand these challenges.
- Most microscopic assessments have been performed at high and constant moisture levels. However, such conditions are not frequent in practice. Understanding the role of low moisture levels and the impact of cyclic conditions on the microstructural properties of ASR-affected concrete is crucial.
- Moisture and temperature have been confirmed to exacerbate ASR in concrete. However, the damage responses and the reduction in mechanical properties over a large range of moisture and temperature conditions and how these responses change over time are unknown. Carrying out more research in this area would improve our understanding of the internal behaviour of concrete at different exposure conditions.

## Figures and Tables

**Table 1:** Moisture measurement techniques.

Test	NDT	Location	Influencing Factor	Output
Gamma densitometry [47]	Yes	Different depth	Geometry	Density of concrete
Electrical resistance [48]	Yes	Surface	Degree of maturity	Electrical conductivity
Hygrometry [49]	Yes	Location of choice	Calibration, temperature	Relative humidity
Chemical reactions [50]	No	Representative sample	Evaporation	Reaction with water
Thermalized neutrons [51]	Yes	Surface	Chemical composition	Thermal neutron detector
Microwave absorption [52]	Yes	Few inches deep	Mix proportion	Electromagnetic waves
Infrared thermography [53]	Yes	No contact	Concrete density	Surface temperature
Gravimetric [54]	No	Representative sample	Depth of collection, incomplete drying	Surface temperature

**Table 2:** Database of ASR expansion at different temperatures.

Ref.	Aggregate Type	Sample Size	Reactive Content	Alkali $\left(Na_{2}O_{eq}\right)$	Temp.	Test Duration	Final Expansion	Expansion at 28 Days
[3]	Highly reactive fine	75 × 75 × 285 mm (prism)	Fine agg.	1.25%	21 °C	167 days	0.14%	0.010%
					40 °C		0.55%	0.020%
[22]	Reactive limestone	16 × 2 cm (cylinder)	Fine agg.	1.84%	60 °C	200 days	0.24%	0.220%
[71]	Highly reactive quartz	75 × 75 × 285 mm (prism)	Coarse agg.	0.925%	38 °C	365 days	0.24%	0.010%
					60 °C	273 days	0.18%	0.040%
[77]	Sudbury	100 × 285 mm (cylinder)	Coarse agg.	1.25%	38 °C	365 days	0.20%	0.010%
					60 °C		0.16%	0.040%
		75 × 75 × 285 mm (prism)			38 °C		0.170%	0.015%
					60 °C		0.080%	0.030%
	Spratt	100 × 285 mm (cylinder)	Coarse agg.	1.25%	38 °C	365 days	0.26%	0.018%
					60 °C		0.18%	0.110%
		75 × 75 × 285 mm (prism)			38 °C		0.21%	0.020%
					60 °C		0.17%	0.100%
[82]	Spratt	75 × 75 × 285 mm (prism)	Coarse agg.	1.25%	38 °C	730 days	0.26%	0.013%
					50 °C	187 days	0.248%	0.085%
[88]	Spratt	75 × 150 mm (cylinder)	Fine agg.	1.07%	38 °C	365 days	0.160%	0.033%
					60 °C		0.074%	0.058%
	Springhill				38 °C		0.230%	0.028%
					60 °C		0.103%	0.050%
[90]	Spratt	75 × 75 × 285 mm (prism)	Coarse agg.	1.25%	38 °C	365 days	0.257%	-------
					60 °C	91 days	0.160%	-------
	Sudbury				38 °C	365 days	0.171%	-------
					60 °C	91 days	0.138%	-------
[91]	Spratt	75 × 75 × 285 mm (prism)	Coarse agg.	1.25%	38 °C	365 days	0.229%	-------
					60 °C	182 days	0.167%	0.103%
	Sudbury				38 °C	365 days	0.150%	-------
					60 °C	182 days	0.187%	0.022%
[92]	Limestone	75 × 75 × 285 mm (prism)	Coarse agg.	1.25%	38 °C	365 days	0.22%	0.000%
					60 °C	150 days	0.19%	0.065%
[93]	Highly reactive andesite	75 × 75 × 250 mm (prism)	Coarse agg.	0.94%	40 °C	365 days	0.140%	0.035%
					60 °C		0.110%	0.070%
[94]	Highly reactive Jobe sand	25 × 25 × 285 mm (prism)	Fine agg.	1.04%	38 °C	600 days	0.800%	0.050%
					55 °C		0.790%	0.350%
[95]	Silica sand and Pyrex glass	28 × 28 × 180 mm (prism)	Fine agg.	1.20%	30 °C	100 days	0.650%	0.350%
					60 °C		0.420%	0.410%

**Table 3:** Microstructure assessment of ASR.

Ref.	Study	Test and Methods	Findings
[38]	Characterization of amorphous and crystalline ASR products formed in concrete aggregates	SEM, X-ray spectroscopy, and Raman microscopy	The morphology of crystalline ASR products is influenced by temperature.
[82]	The effect of elevated conditioning temperature on the ASR expansion, cracking, and properties of reactive Spratt aggregate concrete	Damage rating index	Similar ASR-induced expansion can result in different levels of damage as temperature increases.
[105]	Failure criteria and microstructure evolution mechanism of the alkali–silica reaction of concrete	Scanning electron microscopy (SEM) and X-ray computed microtomography	Microcracks propagate from voids in the aggregate into the cement paste. Presence of ASR gel in the pores leads to a reduction in porosity.
[106]	Quantitative analysis of the evolution of ASR products and crack networks in the context of the concrete mesostructure	Time-lapse X-ray tomography	Visualization of the movement of ASR products from the aggregates into the cement paste in 4D.
[107]	Diagnosis of ASR damage in highway pavement after 15 years of service in wet–freeze climate region	Thin section, SEM, and electron dispersive spectroscopy (EDS)	Cracks were identified in the grains of coarse quartzite aggregate. Gel-like products in the cracks were confirmed to be ASR products by EDS.
[108]	Assessment of the alkali–silica reactivity potential in granitic rocks	X-ray diffraction (XRD) and SEM/EDS	Contents of strained quartz in quartz were determined using image analysis and correlated with AMBT results. There existed a linear correlation between the two observations.
[109]	Composition of alkali–silica reaction products at different locations within concrete structures	Thin section and SEM/EDS	Structure and qualitative properties of ASR gel in different structures were verified. Composition of gel varied with location. Gels that had propagated into the cement paste contained a higher amount of calcium than those in the aggregates.
[110]	Application of electron backscatter diffraction to evaluate the ASR risk of concrete aggregates	SEM and electron backscatter diffraction (EBSD)	The dissolution of quartz at high pH was observed to occur along its grain and sub-grain boundaries. This technique can be used to assess the properties of slow-late reactive aggregates.
[111]	Microstructure, crystallinity, and composition of alkali–silica reaction products in concrete determined by transmission electron microscopy	SEM, EDS, focused ion beam (FIB), and transmission electron microscopy (TEM)	The morphology of ASR products differs with location. Products located in thin grains are amorphous while those in larger widths are crystalline.

**Table 4:** Damage features used in damage rating index [32].

Features	Weighting Factors
CCA: Closed cracks in aggregates	0.25
OCA: Open cracks in aggregates	2
OCAG: Open cracks in aggregates with reaction products	2
CAD: Coarse aggregate debonded	3
DAP: Disaggregated/corroded aggregate particle	2
CCP: Cracks in cement paste	3
CCPG: Cracks in cement paste with reaction products	3

## Data Availability

Not applicable.

## References

[1] Rajabipour F., Giannini E., Dunant C., Ideker J.H., Thomas M.D.A. (2015). Alkali–Silica Reaction: Current Understanding of the Reaction Mechanisms and the Knowledge Gaps. Cem. Concr. Res..

[2] Tragardh J., Lagerblad B. Influence of ASR Expansion on the Frost Resistance of Concrete. Proceedings of the 10th International Conference on Alkali Aggregate Reaction in Concrete.

[3] Deschenes R.A., Giannini E., Drimalas T., Fournier B., Hale W.M. (2018). Effects of Moisture, Temperature, and Freezing and Thawing on Alkali-Silica Reaction. ACI Mater. J..

[4] Boddy A.M., Hooton R.D., Thomas M.D.A. (2003). The Effect of the Silica Content of Silica Fume on Its Ability to Control Alkali–Silica Reaction. Cem. Concr. Res..

[5] Diamond S. (1981). Effects of Two Danish Flyashes on Alkali Contents of Pore Solutions of Cement-Flyash Pastes. Cem. Concr. Res..

[6] Duchesne J., Bérubé M.A. (1994). The Effectiveness of Supplementary Cementing Materials in Suppressing Expansion Due to ASR: Another Look at the Reaction Mechanisms Part 2: Pore Solution Chemistry. Cem. Concr. Res..

[7] Ramlochan T., Thomas M., Gruber K.A. (2000). The Effect of Metakaolin on Alkali–Silica Reaction in Concrete. Cem. Concr. Res..

[8] Venkatachalam S., Raja K., Vishnuvardhan K., Suchithra S., Maniarasan S.K., Saravanan M.M., Miruna M., Prabanjan S. (2022). The ASR Mechanism in Concrete and the Influence of Lithium in Mitigating It: A Critical Review. Mater. Today Proc..

[9] Kaladharan G., Szeles T., Stoffels S.M., Rajabipour F. (2021). Novel Admixtures for Mitigation of Alkali-Silica Reaction in Concrete. Cem. Concr. Compos..

[10] Feng X., Thomas M.D.A., Bremner T.W., Balcom B.J., Folliard K.J. (2005). Studies on Lithium Salts to Mitigate ASR-Induced Expansion in New Concrete: A Critical Review. Cem. Concr. Res..

[11] Leemann A., Lörtscher L., Bernard L., Le Saout G., Lothenbach B., Espinosa-Marzal R.M. (2014). Mitigation of ASR by the Use of LiNO_3_—Characterization of the Reaction Products. Cem. Concr. Res..

[12] Diaz-Loya I., Juenger M., Seraj S., Minkara R. (2019). Extending Supplementary Cementitious Material Resources: Reclaimed and Remediated Fly Ash and Natural Pozzolans. Cem. Concr. Compos..

[13] Oey T., La Plante E.C., Falzone G., Hsiao Y.-H., Wada A., Monfardini L., Bauchy M., Bullard J.W., Sant G. (2020). Calcium Nitrate: A Chemical Admixture to Inhibit Aggregate Dissolution and Mitigate Expansion Caused by Alkali-Silica Reaction. Cem. Concr. Compos..

[14] Xiao R., Huang B., Zhou H., Ma Y., Jiang X. (2022). A State-of-the-Art Review of Crushed Urban Waste Glass Used in OPC and AAMs (Geopolymer): Progress and Challenges. Clean. Mater..

[15] Bérubé M.-A., Chouinard D., Pigeon M., Frenette J., Rivest M., Vézina D. (2002). Effectiveness of Sealers in Counteracting Alkali–Silica Reaction in Highway Median Barriers Exposed to Wetting and Drying, Freezing and Thawing, and Deicing Salt. Can. J. Civ. Eng..

[16] Lute R.D., Folliard K.J., Drimalas T., Rust C.K. Coatings and Sealers for Mitigation of Alkali-Silica Reaction And/or Delayed Ettringite Formation. Proceedings of the 15th International Conference on Alkali-Aggregate Reaction–ICAAR.

[17] Murray C.D. (2014). Durability of Silane Sealer in a Highly Alkaline Environment. Bachelor’s Thesis.

[18] Schindler A., Johnson D., Warnock R., Barnes R. (2018). Effectiveness of Silane to Mitigate Alkali-Silica Reaction in a Historical Bridge. MATEC Web Conf..

[19] Thomas M., Folliard K., Fournier B., Rivard P., Drimalas T. (2013). Methods for Evaluating and Treating ASR-Affected Structures: Results of Field Application and Demonstration Projects Volume I: Summary of Findings and Recommendations Final Report.

[20] Ludwig U. Effects of Environmental Conditions on Alkali-Aggregate Reaction. Proceedings of the 8th International Conference on Alkali-Aggregate Reaction.

[21] Stark D. (1991). The Moisture Condition of Field Concrete Exhibiting Alkali–Silica Reactivity. Proceedings of the Second International Conference on Durability of Concrete.

[22] Poyet S., Sellier A., Capra B., Thèvenin-Foray G., Torrenti J.-M., Tournier-Cognon H., Bourdarot E. (2006). Influence of Water on Alkali-Silica Reaction: Experimental Study and Numerical Simulations. J. Mater. Civ. Eng..

[23] Pedneault A. (1996). Development of Testing and Analytical Procedures for the Evaluation of the Residual Potential of Reaction, Expansion, and Deterioration of Concrete Affected by ASR. Master’s Thesis.

[24] Kurihara T., Katawaki K. Effects of Moisture Control and Inhibition on Alkali Silica Reaction. Proceedings of the 8th International Conference on Alkali-Aggregate Reaction.

[25] Tomosawa F., Tamura K., Abe M. Influence of Water Content of Concrete on Alkali-Aggregate Reaction. Proceedings of the 8th International Conference on Alkali-Aggregate Reaction.

[26] Stark D., Okamoto P., Diamond S. (1993). Eliminating or Minimizing Alkali-Silica Reaktivity.

[27] Sanchez L., Fournier B., Drimalas T., Bastien J., Mitchell D., Noel M. Semi-Quantitative Condition Assessment of Concrete Distress through the Damage Rating Index. Proceedings of the 15th International Conference on Alkali-Aggregate Reaction.

[28] Sanchez L.F.M., Drimalas T., Fournier B. (2020). Assessing Condition of Concrete Affected by Internal Swelling Reactions (ISR) through the Damage Rating Index (DRI). Cement.

[29] Owsiak Z., Zapała-Sławeta J., Czapik P. (2015). Diagnosis of Concrete Structures Distress Due to Alkali-Aggregate Reaction. Bull. Pol. Acad. Sci. Tech. Sci..

[30] Juliana M.M.d.F., Vanessa Karla Barbosa d.S., Deborah Grasielly Cipriano d.S., Dione Luiza d.S., Eliana Cristina B.M. (2018). Alkali-Aggregate Reaction: Definition, Influence and Control. Eng. Appl. Sci..

[31] Du H., Tan K.H. (2014). Effect of Particle Size on Alkali–Silica Reaction in Recycled Glass Mortars. Constr. Build. Mater..

[32] Sanchez L., Fournier B., Jolin M., Duchesne J. (2015). Reliable Quantification of AAR Damage through Assessment of the Damage Rating Index (DRI). Cem. Concr. Res..

[33] Fernandes I., Broekmans M.A.T.M. (2013). Alkali–Silica Reactions: An Overview. Part I. Metallogr. Microstruct. Anal..

[34] Mohammadi A., Ghiasvand E., Nili M. (2020). Relation between Mechanical Properties of Concrete and Alkali-Silica Reaction (ASR); A Review. Constr. Build. Mater..

[35] Hou X., Struble L.J., Kirkpatrick R.J. (2004). Formation of ASR Gel and the Roles of C-S-H and Portlandite. Cem. Concr. Res..

[36] Wang H., Gillott J.E. (1991). Mechanism of Alkali-Silica Reaction and the Significance of Calcium Hydroxide. Cem. Concr. Res..

[37] Frýbort A., Všianský D., Štulířová J., Stryk J., Gregerová M. (2018). Variations in the Composition and Relations between Alkali-Silica Gels and Calcium Silicate Hydrates in Highway Concrete. Mater. Charact..

[38] Leemann A., Shi Z., Lindgård J. (2020). Characterization of Amorphous and Crystalline ASR Products Formed in Concrete Aggregates. Cem. Concr. Res..

[39] Powers T.C., Steinour H.H. (1955). An Interpretation of Some Published Researches on the Alkali–Aggregate Reaction; Part 1—The Chemical Reactions and Mechanisms of Expansion. J. Am. Concr. Inst..

[40] Chatterji S., Jensen A.D., Thaulow N., Christensen P., Denmark T. (1986). Studies of Alkali–Silica Reaction. Part 3. Mechanism by Which NaCl and Ca (OH)_2_ Affect the Reaction. Cem. Concr. Res..

[41] Cai Y., Xuan D., Poon C.S. (2019). Effects of Nano-SiO2 and Glass Powder on Mitigating Alkali-Silica Reaction of Cement Glass Mortars. Constr. Build. Mater..

[42] Gause G.R., Tucker J. (1940). Method for Determining the Moisture Condition in Hardened Concrete. J. Res. Natl. Bur. Stan..

[43] González J.A., López W., Rodríguez P. (1993). Effects of Moisture Availability on Corrosion Kinetics of Steel Embedded in Concrete. Corrosion.

[44] Chen X., Huang W., Zhou J. (2012). Effect of Moisture Content on Compressive and Split Tensile Strength of Concrete. Indian J. Eng. Mater. Sci..

[45] Lura P., Winnefeld F., Fang X. (2017). A Simple Method for Determining the Total Amount of Physically and Chemically Bound Water of Different Cements. J. Therm. Anal. Calorim..

[46] Hundt J., Buschmann J. (1971). Moisture Measurement in Concrete: Analysis of the Results of a RILEM Inquiry Carried out by the B.A.M. Mat. Constr..

[47] Kumara W.A.S., Halvorsen B.M., Melaaen M.C. (2010). Single-Beam Gamma Densitometry Measurements of Oil–Water Flow in Horizontal and Slightly Inclined Pipes. Int. J. Multiph. Flow.

[48] De Jong S.M., Heijenk R.A., Nijland W., van der Meijde M. (2020). Monitoring Soil Moisture Dynamics Using Electrical Resistivity Tomography under Homogeneous Field Conditions. Sensors.

[49] Andrade C., Sarría J., Alonso C. (1999). Relative Humidity in the Interior of Concrete Exposed to Natural and Artificial Weathering. Cem. Concr. Res..

[50] Nilsson L.-O. (2018). Methods of Measuring Moisture in Building Materials and Structures.

[51] Zeilinger A., Hübner R. (1975). Measurement of Moisture Motion Under a Temperature Gradient in a Concrete for SNR-300 Using Thermal Neutrons. Proceedings of the Concrete Properties Relevant to PCRV.

[52] Pandey T., Bhuiya T., Singh B., Harsh R. (2012). A Review on Microwave Based Moisture Measurement System for Granular Materials. IOSR J. Electron. Commun. Eng. (IOSR-JECE).

[53] Grinzato E., Ludwig N., Cadelano G., Bertucci M., Gargano M., Bison P. (2011). Infrared Thermography for Moisture Detection: A Laboratory Study and In-Situ Test. Mater. Eval..

[54] Derome D., Fazio P. (1998). Experimental Setup for the Study of Air Leakage Patterns. Proceedings of the Thermal Performance of the Exterior Envelopes of Buildings VII.

[55] Lindgård J., Rodum E., Pedersen B. (2006). Alkali-Silica Reactions in Concrete—Relationship between Water Content and Observed Damage on Structures. ACI Symp. Publ..

[56] Yang Q. (1999). Inner Relative Humidity and Degree of Saturation in High-Performance Concrete Stored in Water or Salt Solution for 2 Years. Cem. Concr. Res..

[57] Zhang J., Wang J., Han Y. (2015). Simulation of Moisture Field of Concrete with Pre-Soaked Lightweight Aggregate Addition. Constr. Build. Mater..

[58] Zhang J., Gao Y., Han Y., Sun W. (2012). Shrinkage and Interior Humidity of Concrete under Dry–Wet Cycles. Dry. Technol..

[59] Geiker M.R., Laugesen P. (2001). On the Effect of Laboratory Conditioning and Freeze/Thaw Exposure on Moisture Profiles in HPC. Cem. Concr. Res..

[60] Lindgård J., Andiç-Çakır Ö., Fernandes I., Rønning T.F., Thomas M.D.A. (2012). Alkali–Silica Reactions (ASR): Literature Review on Parameters Influencing Laboratory Performance Testing. Cem. Concr. Res..

[61] Wardeh G., Perrin B. (2006). Relative Permeabilities of Cement-Based Materials: Influence of the Tortuosity Function. J. Build. Phys..

[62] Chaudhry R.H. (2018). Determination of Air Voids, Capillary, and Gel Porosity in Hardened Concrete Using Mass-Based Saturation Techniques. Master’s Thesis.

[63] Weiss J. Relating Transport Properties to Performance in Concrete Pavements; Map Brief. CP Road Map. December 2014. National Concrete Pavement Technology Center, Ames, IA. https://intrans.iastate.edu/app/uploads/2018/08/MAPbriefDecember2014.pdf.

[64] Olajide O., Nokken M., Sanchez L. A Review on the Role of Moisture and Temperature in Alkali-Silica Reaction (ASR). Proceedings of the 16th International Conference on Alkali-Aggregate Reaction in Concrete (ICAAR).

[65] Saccani A., Bonora V., Monari P. (2001). Laboratory Short-Term Evaluation of ASR: A Contribution. Cem. Concr. Res..

[66] Lenzner D. Influence of the Amount of Mixing Water on the Alkali-Silica Reaction. Proceedings of the 5th International Conference on Alkali-Aggregate Reaction.

[67] Nilsson L.-O., Peterson O. (1983). A Moisture Problem Causing Pop-Outs in Concrete Floors.

[68] Multon S., Toutlemonde F. (2010). Effect of Moisture Conditions and Transfers on Alkali Silica Reaction Damaged Structures. Cem. Concr. Res..

[69] Multon S., Seignol J.-F., Toutlemonde F. (2005). Structural Behavior of Concrete Beams Affected by Alkali-Silica Reaction. ACI Mater. J..

[70] Olafsson H. The Effect of Relative Humidity and Temperature on Alkali Expansion of Mortar Bars. Proceedings of the 7th International Conference on Alkali Aggregate Reaction in Concrete.

[71] Lindgård J., Thomas M.D.A., Sellevold E.J., Pedersen B., Andiç-Çakır Ö., Justnes H., Rønning T.F. (2013). Alkali–Silica Reaction (ASR)—Performance Testing: Influence of Specimen Pre-Treatment, Exposure Conditions and Prism Size on Alkali Leaching and Prism Expansion. Cem. Concr. Res..

[72] Bažant Z.P., Steffens A. (2000). Mathematical Model for Kinetics of Alkali–Silica Reaction in Concrete. Cem. Concr. Res..

[73] Hashemi A., Donnell K.M., Zoughi R., Kurtis K.E. (2015). Effect of Humidity on Dielectric Properties of Mortars with Alkali-Silica Reaction (ASR) Gel. Proceedings of the 2015 IEEE International Instrumentation and Measurement Technology Conference (I2MTC) Proceedings.

[74] BCA (1992). The Diagnosis of Alkali–Silica Reaction-Report of a Working Party.

[75] Reed R.G. (2016). Measuring Relative Humidity in Concrete Pavements as a Method to Assess ASR Mitigation Measures. Bachelor’s Thesis.

[76] Gudmundsson B., Asgeirsson H. (1975). Some Investigations on Alkali Aggregate Reaction. Cem. Concr. Res..

[77] Sinno N., Shehata M.H. (2021). Role of Temperature on Alkali-Silica Reaction and the Efficacy of Supplementary Cementitious Materials. Constr. Build. Mater..

[78] Guo J.-J., Liu P.-Q., Wu C.-L., Wang K. (2021). Effect of Dry–Wet Cycle Periods on Properties of Concrete under Sulfate Attack. Appl. Sci..

[79] Farny J.A., Kerkhoff B. (2007). Diagnosis and Control of Alkali-Aggregate Reactions in Concrete.

[80] Kagimoto H., Kawamura M. (2011). Measurements of Strain and Humidity within Massive Concrete Cylinders Related to the Formation of ASR Surface Cracks. Cem. Concr. Res..

[81] Kagimoto H., Yasuda Y., Kawamura M. Effects of Expansion Behavior of ASR-Affected Concrete in Atmospheres with Various Values of Relative Humidity on Surface Cracking. Proceedings of the 15th International Conference on Alkali Aggregate Reaction in Concrete.

[82] Gautam B.P., Panesar D.K. (2017). The Effect of Elevated Conditioning Temperature on the ASR Expansion, Cracking and Properties of Reactive Spratt Aggregate Concrete. Constr. Build. Mater..

[83] Deschenes R.A. (2017). Mitigation and Evaluation of Alkali-Silica Reaction (ASR) and Freezing and Thawing in Concrete Transportation Structures. Theses Diss..

[84] Gillott J.E. (1975). Alkali-Aggregate Reactions in Concrete. Eng. Geol..

[85] Helmuth R., Stark D., Diamond S., Moranville-regourd M. Alkali-Silica Reactivity: An Overview of Research. Proceedings of the National Research Council.

[86] Lu D., Zhou X., Xu Z., Lan X., Tang M., Fournier B. (2006). Evaluation of Laboratory Test Method for Determining the Potential Alkali Contribution from Aggregate and the ASR Safety of the Three-Gorges Dam Concrete. Cem. Concr. Res..

[87] Bérubé M.-A., Duchesne J., Dorion J.F., Rivest M. (2002). Laboratory Assessment of Alkali Contribution by Aggregates to Concrete and Application to Concrete Structures Affected by Alkali–Silica Reactivity. Cem. Concr. Res..

[88] Drolet C., Duchesne J., Fournier B. (2017). Effect of Alkali Release by Aggregates on Alkali-Silica Reaction. Constr. Build. Mater..

[89] Maia Neto F.M., Andrade T.W.C.O., Gomes R.M., Leal A.F., Almeida A.N.F., Lima Filho M.R.F., Torres S.M. (2021). Considerations on the Effect of Temperature, Cation Type and Molarity on Silica Degradation and Implications to ASR Assessment. Constr. Build. Mater..

[90] Ideker J.H., East B.L., Folliard K.J., Thomas M.D.A., Fournier B. (2010). The Current State of the Accelerated Concrete Prism Test. Cem. Concr. Res..

[91] Fournier B., Chevrier R., Grosbois M., Lisella R., Folliard K., Ideker J., Shehatad M., Thomas M., Baxter S. (2014). The Accelerated Concrete Prism Test (60 °C): Variability of the Test Method and Proposed Expansion Limits. Researchgate.

[92] Sanchez L., Kuperman S.C., Helene P. (2011). Using the Accelerated Brazilian Concrete Prism Test (ABCPT) to Evaluate Alkali Aggregate Reaction (AAR). IBRACON Struct. Mater. J..

[93] Kawabata Y., Dunant C., Yamada K., Scrivener K. (2019). Impact of Temperature on Expansive Behavior of Concrete with a Highly Reactive Andesite Due to the Alkali–Silica Reaction. Cem. Concr. Res..

[94] Kim T., Olek J., Jeong H. (2015). Alkali–Silica Reaction: Kinetics of Chemistry of Pore Solution and Calcium Hydroxide Content in Cementitious System. Cem. Concr. Res..

[95] Li B., Wang Z.-R., Liu H.-B., Liu X.-Z., Li H., Chen X. (2019). Meso-Mechanical Research on Alkali-Silica Reaction Expansion in Pyrex Glass and Silica Sand at Different Temperatures and Curing Times. Constr. Build. Mater..

[96] Folliard K.J., Ideker J.H., Thomas M.D., Fournier B. Assessing Aggregate Reactivity Using the Accelerated Concrete Prism Test. Proceedings of the 7th CANMET/ACI International Conference on Recent Advances in Concrete Technology.

[97] Li B., Baingam L., Kurumisawa K., Nawa T., Liu X.Z. (2018). Micro-Mechanical Modelling for the Prediction of Alkali-Silica Reaction (ASR) Expansion: Influence of Curing Temperature Conditions. Constr. Build. Mater..

[98] Golmakani F., Hooton R.D. (2019). Impact of Pore Solution Concentration on the Accelerated Mortar Bar Alkali-Silica Reactivity Test. Cem. Concr. Res..

[99] Kawabata Y., Yamada K., Sagawa Y., Ogawa S. (2018). Alkali-Wrapped Concrete Prism Test (AW-CPT)—New Testing Protocol toward a Performance Test against Alkali-Silica Reaction–. J. Adv. Concr. Technol..

[100] Lindgård J., Nixon P.J., Borchers I., Schouenborg B., Wigum B.J., Haugen M., Åkesson U. (2010). The EU “PARTNER” Project—European Standard Tests to Prevent Alkali Reactions in Aggregates: Final Results and Recommendations. Cem. Concr. Res..

[101] De Grazia M.T., Goshayeshi N., Gorga R., Sanchez L.F.M., Santos A.C., Souza D.J. (2021). Comprehensive Semi-Empirical Approach to Describe Alkali Aggregate Reaction (AAR) Induced Expansion in the Laboratory. J. Build. Eng..

[102] Cukierski D. Quantifying Alkali-Silica Reaction in Concrete: Damage Rating Index. Proceedings of the Concrete Institute of Australia’s Biennial National Conference.

[103] Rivard P., Ballivy G. (2005). Assessment of the Expansion Related to Alkali-Silica Reaction by the Damage Rating Index Method. Constr. Build. Mater..

[104] Sanchez L., Fournier B., Jolin M., Sanchez L.F.M., Fournier B., Jolin M., Bustamante M.A.B. Evaluation of the Microscopic ASR Features through the Damage Rating Index (DRI) for Different Concrete Strengths and Aggregate Types (Fine and Coarse Reactive Aggregates). Proceedings of the 14th Euroseminar on Microscopy Applied to Building Materials.

[105] Wang Y., Gao P., Su H., Qin Y., Wang Y., Xue G. (2023). Failure Criteria and Microstructure Evolution Mechanism of the Alkali–Silica Reaction of Concrete. Rev. Adv. Mater. Sci..

[106] Shakoorioskooie M., Griffa M., Leemann A., Zboray R., Lura P. (2022). Quantitative Analysis of the Evolution of ASR Products and Crack Networks in the Context of the Concrete Mesostructure. Cem. Concr. Res..

[107] Glinicki M.A., Jóźwiak-Niedźwiedzka D., Antolik A., Dziedzic K., Dąbrowski M., Bogusz K. (2022). Diagnosis of ASR Damage in Highway Pavement after 15 Years of Service in Wet-Freeze Climate Region. Case Stud. Constr. Mater..

[108] Antolik A., Jóźwiak-Niedźwiedzka D. (2021). Assessment of the Alkali-Silica Reactivity Potential in Granitic Rocks. Constr. Build. Mater..

[109] Fernandes I. (2009). Composition of Alkali–Silica Reaction Products at Different Locations within Concrete Structures. Mater. Charact..

[110] Rößler C., Möser B., Giebson C., Ludwig H.-M. (2017). Application of Electron Backscatter Diffraction to Evaluate the ASR Risk of Concrete Aggregates. Cem. Concr. Res..

[111] Boehm-Courjault E., Barbotin S., Leemann A., Scrivener K. (2020). Microstructure, Crystallinity and Composition of Alkali-Silica Reaction Products in Concrete Determined by Transmission Electron Microscopy. Cem. Concr. Res..

[112] Sanchez L.F.M., Drimalas T., Fournier B., Mitchell D., Bastien J. (2018). Comprehensive Damage Assessment in Concrete Affected by Different Internal Swelling Reaction (ISR) Mechanisms. Cem. Concr. Res..

[113] Joo H.E., Takahashi Y. (2023). Analytical and Experimental Studies on Alkali-Silica Reaction Mechanism: Aggregate Cracking and Chemical Composition Change of Gel. Cem. Concr. Compos..

